# Quantum Cascade Laser-Based Photoacoustic Spectroscopy for Trace Vapor Detection and Molecular Discrimination

**DOI:** 10.3390/s100301986

**Published:** 2010-03-11

**Authors:** Ellen Holthoff, John Bender, Paul Pellegrino, Almon Fisher

**Affiliations:** 1 United States Army Research Laboratory, RDRL-SEE-O, 2800 Powder Mill Road, Adelphi, MD 20783, USA; E-Mails: john.s.bender@us.army.mil (J.B.); ppellegr@arl.army.mil (P.P.); 2 Infotonics Technology Center, 5450 Campus Drive, Canandaigua, NY 14424, USA; E-Mail: almon.fisher@ITCMEMS.com

**Keywords:** photoacoustic spectroscopy, sensor, quantum cascade laser, MEMS, chemometrics

## Abstract

We report on the development of a microelectromechanical systems (MEMS)-scale photoacoustic sensor for the detection of trace gases. A mid-infrared quantum cascade laser (QCL) was used to determine detection limits for acetic acid, acetone, 1,4-dioxane, and vinyl acetate. The source was continuously tunable from 1015 cm^−1^ to 1240 cm^−1^, allowing for the collection of photoacoustic vibrational spectra for these gases. Exceptional agreement between the measured photoacoustic spectra and the infrared spectra for acetic acid, acetone, 1,4-dioxane, and vinyl acetate was observed. Partial least-squares (PLS) regression was used to develop an algorithm for classification of these compounds based solely on photoacoustic spectra.

## 1. Introduction

Monitoring trace gases is of great importance in a wide range of applications. The Global War on Terror has made rapid detection and identification of chemical and biological agents a priority for military and homeland defense applications; while escalating environmental awareness has led to more restrictive industrial regulations on air quality. Reliable real-time detection of these threats is complicated by a diverse range of materials. Both the military and industry have expressed interest in the development of more sensitive and adaptable trace gas analysis equipment.

Photothermal spectroscopy encompasses a group of highly sensitive methods that can be used to detect trace levels of gases using optical absorption and subsequent thermal perturbations of the gases. The underlying principle that connects these various spectroscopic methods is the measurement of physical changes (*i.e.*, temperature, density, or pressure) as a result of photo-induced change in the thermal state of the sample. In general, photothermal methods are classified as indirect methods for detection of trace optical absorbance, because the transmission of the light used to excite the sample is not measured directly. Examples of photothermal techniques include photothermal interferometry (PTI), photothermal lensing (PTL), photothermal deflection (PTD), and photoacoustic spectroscopy (PAS). In comparison to other photothermal techniques, which measure the refractive index using combinations of probe sources and detectors, PAS measures the pressure wave produced by sample heating. Previous research suggests that PAS is a particularly sensitive technique, capable of trace gas detection at parts-per-trillion (ppt) levels. [1,2] Although these studies demonstrate the sensitivity capabilities of photoacoustic sensors, the total system size represents a large logistics burden in terms of size, cost, and power consumption.

To date, limited research has been done to demonstrate the feasibility of a miniaturized photoacoustic sensor [3–9]. Initial examination of the scaling principles associated with PAS in respect to microelectromechanical systems (MEMS) dimensions indicated that photoacoustic signals would remain at similar sensitivities or even surpass those commonly found in macro-scale devices [3–8]. More recently, we demonstrated the use of both CO_2_ laser and quantum cascade laser (QCL)-based MEMS-scale photoacoustic sensors to provide detection limits at parts-per-billion (ppb) levels for the nerve gas simulant, dimethyl methyl phosphonate (DMMP). [8,10] The 100 cm^−1^ continuous tuning range of the QCL employed in the QCL-based sensor allowed for the photoacoustic vibrational spectrum of DMMP to be collected. This spectrum was in agreement with the infrared spectrum of the simulant. These results suggest that combining a continuously tunable QCL having a broad tuning range with a MEMS-scale photoacoustic device would provide increased molecular discrimination as well as simultaneous detection of several molecules of interest. In this current work, we report on the use of a continuously tunable QCL having a tuning range of 225 cm^−1^, in combination with a MEMS-scale photoacoustic cell design, for detection and discrimination of acetic acid, acetone, 1,4-dioxane, and vinyl acetate. These compounds were chosen specifically for this proof of concept demonstration because each has known absorbance features in the wavelength tuning range of the QCL. To our knowledge, this is the first reported study dedicated to the development of a miniaturized photoacoustic sensor employing a single, continuously tunable QCL for the simultaneous detection and discrimination of numerous molecules of interest.

## 2. Experimental Section

**Figure 1.**
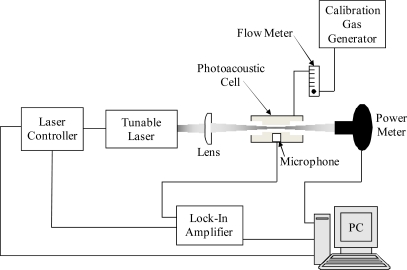
Schematic diagram of a general photoacoustic gas sensor system.

Figure 1 depicts a block diagram of the basic elements required for a photoacoustic gas sensor. In order to generate acoustic waves in gases, periodic heating and cooling of the sample is required to produce pressure fluctuations. This is accomplished using modulated or pulsed excitation sources [11–13]. The pressure waves detected in PAS are generated directly by the absorbed fraction of the modulated or pulsed excitation beam. Therefore, the signal generated from a photoacoustic experiment is directly proportional to the absorbed incident power.

### 2.1. Quantum Cascade Laser

A broadly tunable, external cavity pulsed QCL (Daylight Solutions 11088) was employed as the excitation source for the sensing system. The laser was powered by an external controller (Daylight Solutions TLC 1001). The QCL was continuously tunable from 1,015 cm^−1^ to 1,240 cm^−1^ and had a spectral resolution of 1 cm^−1^. The pulsed source operated at room temperature with convective cooling. Current pulses of 1,600 mA with a 500 ns duration and 21.6 kHz pulse rate corresponded to a 1.1% duty cycle and provided an average optical power of 1.32 (±0.33) mW. The transmitted laser power was measured with a power meter (Ophir Optronics Nova II) equipped with a thermal head (Ophir Optronics 3A). These measurements allowed for normalization of the photoacoustic signal for any residual drift associated with the excitation source. A BaF_2_ plano-convex lens (ISP Optics BF-PX-12-25) was utilized for focusing purposes.

### 2.2. MEMS-Scale Photoacoustic Cell

A MEMS-scale differential photoacoustic cell was fabricated to meet our design specifications [14] by Infotonics Technology Center, Inc. The differential technique employs two resonator tubes, both housing a microphone (Knowles FG-23629), but with radiation directed only through one to generate a photoacoustic signal. The microphones possessed similar responsivities, which allowed for subtraction of the reference microphone signal from the photoacoustic microphone signal. This allowed for the removal of noise elements that were present in both resonant chambers, such as external vibrations.

**Figure 2.**
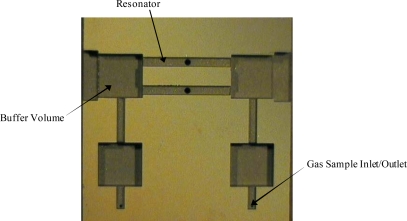
Photograph of the internal structure of the MEMS-scale photoacoustic cell.

The influence of cell geometry on the photoacoustic signal has been discussed elsewhere [2,15]. Briefly, the cell consisted of two 8.5 mm long open resonators having square cross sections, each with a diameter of 0.93 mm. The resonator was flanked on both sides by a buffer volume (acoustic filter), which provided noise suppression. The resonator length was twice that of the buffer volume and the diameter of the buffer volume was at least three times that of the resonator. To further suppress gas flow noise, the buffer volumes were each connected by a tube to gas input and output acoustic filters (Figure 2).

**Figure 3.**
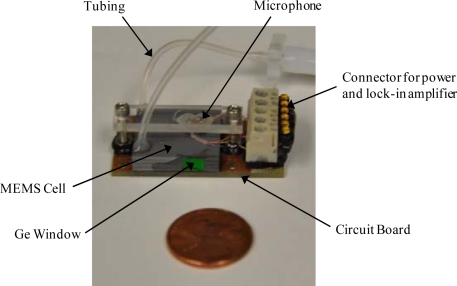
Photograph of the MEMS-scale photoacoustic cell used when collecting data.

The cell had two germanium windows (Edmund Optics NT47-685), which were attached to the buffer volumes on either side of the photoacoustic resonator with epoxy. Tygon^®^ tubing was connected to the buffer volumes to allow for gas sample inlet and outlet flow. The MEMS-scale photoacoustic cell was mounted on a printed circuit board, which allowed for wiring the microphones to a power supply (AA battery) and a lock-in amplifier (via modified BNC cables). Figure 3 is a photograph of the complete MEMS-scale photoacoustic cell package.

### 2.3. Sample Generation

The trace gases were generated using calibration gas generators (Owlstone Nanotech OVG-4 and VICI Metronics Dynacalibrator 190). Nitrogen was used as the carrier gas. The gas sources were gravimetrically certified permeation tubes (KIN-TEK HRT-008.50-3039/40 (acetic acid); HRT-010.00-3026/40 (acetone); HRT-002.00-3045/100 (1,4-dioxane); HRT-007.00-3047/40 (vinyl acetate)). The acetic acid, acetone, and vinyl acetate tubes were placed in calibration ovens held at a constant temperature of 40 °C. The permeation rates at this temperature for acetic acid, acetone, and vinyl acetate were 819 ng/min, 1,114 ng/min, and 1,986 ng/min, respectively. The 1,4-dioxane tube was placed in a calibration oven held at a constant temperature of 100 °C. The permeation rate at this temperature was 1,715 ng/min. Varying calibrated flow rates of the nitrogen carrier gas from 50 mL/min to 1000 mL/min governed the concentration of the analytes of interest. The concentration range for each analyte is limited by the permeation tube and the permissible flow rates of the calibration gas generators. Poly (tetrafluoroethylene) (PTFE) tubing was used to connect the gas generator to the sample inlet of the photoacoustic cell. A flow controller and a relief valve were placed in line between the gas generator and the photoacoustic cell to ensure a constant flow rate of 60 mL/min through the cell. This was done to reduce flow noise.

### 2.4. Data Acquisition

The signals detected by both the photoacoustic and reference microphones were extracted using the differential voltage input on a lock-in amplifier (Stanford Research Systems SR530) with a time constant of 3 s operating at the pulse frequency of the laser. LabVIEW (National Instruments, version 2009) was used to create a virtual instrument (VI) to read and record the voltage outputs from the lock-in amplifier under various conditions directly to a personal computer (PC). The VI was programmed to collect the X (in-phase), Y (quadrature), R (amplitude) and θ (phase angle) components of the photoacoustic signal. Photoacoustic spectra were obtained by holding the laser pulse frequency constant while scanning the laser wavelength range. This allowed for the determination of the frequency having the maximum analyte absorbance in this region. 200 measurements were made at each increment, and a mean value was calculated and recorded for the subsequent construction of a photoacoustic spectrum. For each analyte, the photoacoustic spectrum revealed a constant background signal, attributed to the absorbance of laser radiation by the cell windows and walls. To collect photoacoustic spectra for several sample concentrations, the nitrogen carrier gas flow rate was varied in the calibration gas generator. The maximum photoacoustic absorbance signal recorded for several concentrations was used to prepare a linear regression. The limit of detection (LOD) was calculated by taking three times the standard deviation (3*σ*) of the background signal and dividing it by the slope of the linear function.

### 2.5. Instrumentation

All Fourier transform infrared (FTIR) absorbance spectra were collected using a Thermo Scientific Nicolet 6700 FTIR spectrometer equipped with a potassium bromide (KBr) beamsplitter and a mercury cadmium telluride (MCT)-A (narrow band–650 cm^−1^ cutoff) detector. Each spectrum was acquired at a resolution of 1.0 cm^−1^ averaging 100 scans.

### 2.6. Software

The Unscrambler (version 9.8) multivariate analysis and experimental design software was purchased from CAMO Software (Woodbridge, NJ) and used to develop partial least squares (PLS2) regression algorithms for classification of unknown samples.

## 3. Results and Discussion

### 3.1. Spectroscopic Data

**Figure 4.**
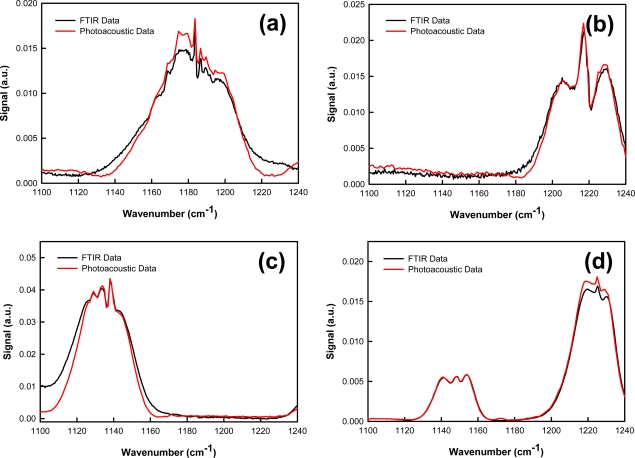
Measured laser photoacoustic spectrum of **(a)** acetic acid; **(b)** acetone; **(c)** 1,4-dioxane; and **(d)** vinyl acetate compared to FTIR reference spectrum.

Laser photoacoustic spectra were collected for acetic acid, acetone, 1,4-dioxane, and vinyl acetate. The intensity-normalized spectra are provided in Figure 4. All spectra were collected as the laser was continuously tuned from 1,100 cm^−1^ to 1,240 cm^−1^ (9.09 μm–8.06 μm), in 1 cm^−1^ increments. These analytes have known absorption features in this region, assigned to carbon—carbon and carbon—oxygen stretching vibrations. [16,17] For all species, there is excellent agreement between the photoacoustic and FTIR spectra.

### 3.2. Sensor Responsivity

**Figure 5.**
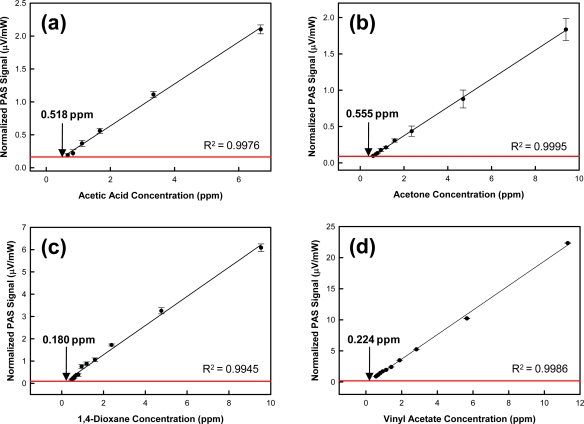
Photoacoustic sensor response as a function of **(a)** acetic acid; **(b)** acetone; **(c)** 1,4-dioxane; and **(d)** vinyl acetate concentration. Error bars represent one standard deviation. The red line represents three standard deviations (3σ) of the background signal. A linear function has been fit to the data.

Photoacoustic sensor limits of detection were determined for acetic acid, acetone, 1,4-dioxane, and vinyl acetate. Figure 5 illustrates the sensor response as a function of analyte concentration measured at the absorbance maximum (relative to the laser wavelength range) for each analyte. The results exhibit excellent linearity. Coefficients of determination (R^2^) can be found in Figure 5. Specific LOD values are also provided in Figure 5.

As a measure of the effectiveness of this sensing platform, we considered the National Institute for Occupational Safety and Health (NIOSH) recommended airborne exposure limits for acetic acid [18], acetone [19], 1,4-dioxane [20], and vinyl acetate [21] (Table 1). In this report, we have achieved detection limits well below the suggested values. These results attest to the effectiveness of this MEMS-scale photoacoustic sensing platform.

### 3.3. Partial Least Squares Regression Analysis

**Figure 6.**
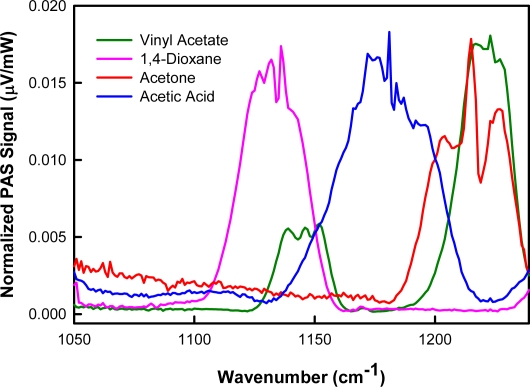
Laser photoacoustic spectral absorption features of acetic acid, acetone, 1,4-dioxane, and vinyl acetate in the 1050 cm^−1^–1240 cm^−1^ (9.52 μm–8.06 μm) region. The spectra have been intensity-normalized.

As illustrated in Figure 6, the spectral region from 1,050 cm^−1^ to 1,240 cm^−1^ is appealing as it contains absorption features representative of vibrational modes present in the selected molecules. It is useful to compare the position of these features in this wavelength range given that the analytes have similar molecular components. In principle, spectral differences among acetic acid, acetone, 1,4-dioxane, and vinyl acetate, such as shape and position of spectral features, make it possible to distinguish these analytes by univariate analysis (*i.e.*, dependent on a single wavelength). However, most univariate approaches do not allow for simultaneous identification of multiple analytes. An alternative approach is multivariate analysis (*i.e.*, full spectral region), which permits simultaneous analysis of multiple components [22].

We have utilized the partial least squares 2 (PLS2) regression method to develop a model for the simultaneous differentiation of acetic acid, acetone, 1,4-dioxane, and vinyl acetate based on their absorbance features (from 1100 cm^−1^–1240 cm^−1^) acquired using QCL-based photoacoustic spectroscopy, including a MEMS-scale photoacoustic cell. We have used training samples having a wide range of analyte concentrations as the basis to develop this model. The model simultaneously uses four algorithms; each devoted to the identification of a particular species (acetic acid, acetone, 1,4-dioxane, or vinyl acetate). It is the concurrent application of the algorithms that facilitates the classification (*i.e.*, identification) of unknown spectra. A model using 1–10 factors or principal components (PCs) was constructed for the prediction of the analytes of interest. Segmented cross validation [23], which included 4 segments of data having 25% of the samples in each segment, was used for model validation. Model diagnostic analyses suggest that the classification model performs well with 4 PCs. The model diagnostics used to establish this number of PCs included percent variance, root mean square error of prediction (RMSEP) plots, and loadings plots [22].

**Figure 7.**
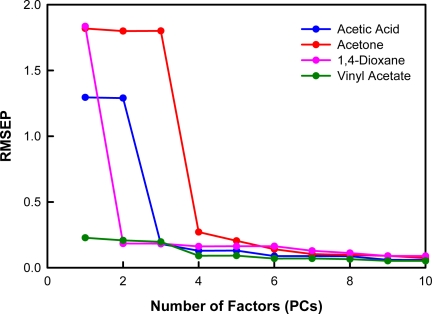
RMSEP *versus* number of factors (PCs) for each analyte of interest in the PLS2 model.

**Figure 8.**
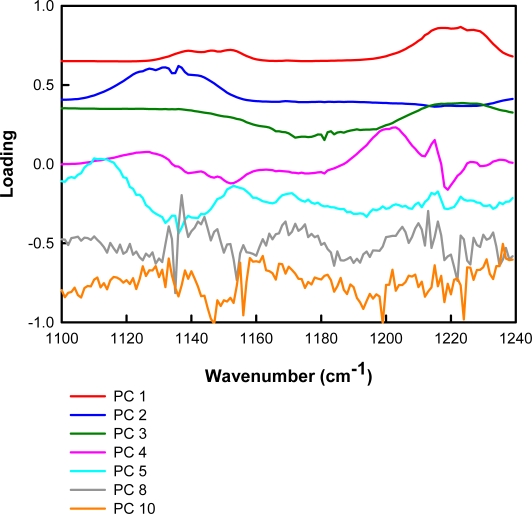
Loadings 1–5, 8, and 10 for the PLS2 model (offset added for clarity).

The concentration and spectral percent variances are given in Table 2. These values correspond to the total variation in analyte concentration and spectral data for all of the analytes of interest. After 4 factors, 99.14% of the concentration variance and 99.98% of the spectral variance are explained. These results are reasonable given that the model is to be used for the differentiation of four different chemical species. The RMSEP plots for the model are shown in Figure 7. Ideally, the RMSEP plots should decrease as factors are added into the model. The optimal number of PCs to include corresponds to the minimum RMSEP value or the point at which the RMSEP value levels off [22]. For this model, the point at which the RMSEP value levels off for all of the analytes of interest occurs when four PCs are included. The loadings for PCs 1–5, 8, and 10 are shown in Figure 8. Although the fifth factor loading contains nonrandom variation, it describes a small amount of spectral variation. The loadings for factors 1–4 are very smooth and have peaks similar to the original data. Therefore, the loadings are not inconsistent with a model having four PCs.

**Figure 9.**
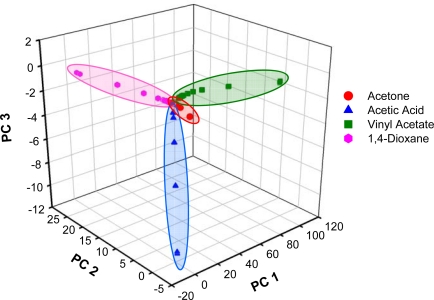
Scores plot for the PLS2 model, using the first three calculated PCs.

Scores for the first three PCs are plotted in Figure 9. As illustrated in the plot, the training spectra for acetic acid, acetone, 1,4-dioxane, and vinyl acetate are defined by four distinct groups. Data points for each analyte are encircled in colored lobes as a guide to the eye and convey no direct information. These results confirm that the spectral features for the four analytes, and therefore their molecular compositions, are different. Furthermore, segregation of the training spectra into four lobes suggests that this model can be used to distinguish these particular species from one another; however the overlapping lobes suggest the potential for an increased false alarm rate at low concentrations.

**Figure 10.**
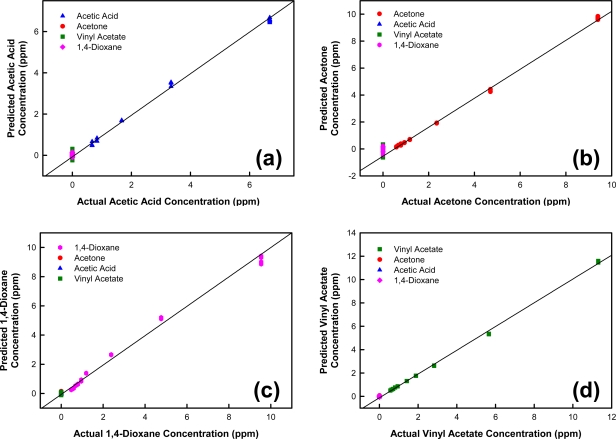
The predicted concentration value plotted against the measured concentration value using **(a)** acetic acid-specific algorithm; **(b)** acetone-specific algorithm; **(c)**1,4-dioxane-specific algorithm; and **(d)** vinyl acetate-specific algorithm. Classification for each analyte is based on four PCs.

As a preliminary validation, this model was used to predict (e.g., classify and quantify) the training spectra used to make the model (cross validation). The predicted results are shown in Figure 10. Figure 10a illustrates the training samples divided into two groups. Samples containing acetic acid are properly identified, based on the predicted acetic acid concentration, by the acetic acid-specific algorithm. Low concentration values (less than the minimum acetic acid concentration used for calibration) are predicted for samples that do not contain acetic acid. These results were expected for these samples as the model is unable to predict reliable analyte concentrations below the minimum calibration concentration. Acetic acid-containing training samples having concentrations in the calibration range are predicted to have concentrations approximately equivalent to the actual concentration of the training sample. Applying the other analyte-specific algorithms for acetone, 1,4-dioxane, and vinyl acetate to an identical training sample set results in similar calibration plots in which acetone (Figure 10b), 1,4-dioxane (Figure 10c), and vinyl acetate (Figure 10d) samples are accurately quantified as containing acetone, 1,4-dioxane, or vinyl acetate, respectively. Low concentration values are predicted for samples that do not contain the specified analyte. These results demonstrate the use of this model to classify the samples as either acetic acid, acetone, 1,4-dioxane, or vinyl acetate, as well as accurately quantify the sample concentration.

The criteria used to assess the quality of the regression for acetic acid, acetone, 1,4-dioxane, and vinyl acetate are provided in Table 3. Calibration and validation slopes and coefficients of determination (R^2^) for each analyte are close to one, and offset values are near zero. These statistics suggest that the prediction quality of the model is excellent. Additionally, small root mean square error of calibration (RMSEC) and root mean square error of prediction (RMSEP) values were shown. RMSEC is the modeling error, calculated from the original training spectra. RMSEP is the average error that can be expected with future predictions. Therefore, the estimated precision of future predictions is 2 × RMSEP. Both RMSEC and RMSEP are expressed in original measurement units (ppm) [23].

**Figure 11.**
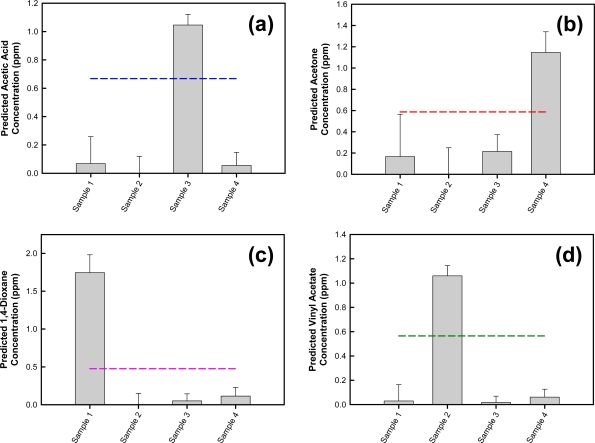
Identification of validation samples using **(a)** acetic acid-specific algorithm; **(b)** acetone-specific algorithm; **(c)**1,4-dioxane-specific algorithm; and **(d)** vinyl acetate-specific algorithm. Classification for each analyte is based on four PCs. The threshold concentration for each analyte is represented by a dotted line.

To better assess its performance, the model was used to predict a new set of photoacoustic spectra acquired using QCL-based photoacoustic spectroscopy, including a MEMS-scale photoacoustic cell. This validation set (Table 4, Samples 1–4) was composed of acetic acid, acetone, 1,4-dioxane, and vinyl acetate sample spectra collected for known analyte concentrations. These spectra were not used to develop the classification algorithm and thus can be used to challenge the model. Prediction results are provided in Figure 11. A threshold concentration was established for each analyte. This delineation represents the minimum concentration of each analyte used for calibration and therefore the lower boundary of the modeled range. In principle, concentration values above this threshold are indicative of analyte identification, while values below this threshold suggest the sample (*i.e.*, photoacoustic spectrum) is not comparable to the presumed species. Error bars signify deviations, which are uncertainty limits given to predicted concentration values. These limits are calculated by the software from the validation variances, the residual variances, and the leverage of the X data (*i.e.*, photoacoustic data) in the prediction samples. Leverage is a measure of how extreme a data point is compared to the majority. Therefore, if the photoacoustic data for the prediction samples are similar to the photoacoustic data for the training samples, then the deviation interval will be smaller and the prediction more reliable. Likewise, if the prediction sample data is different from the training data, the deviation interval will be larger [23].

As shown in Figure 11a, the acetic acid-specific algorithm applied to this sample set predicts an acetic acid concentration greater than the threshold value for the validation acetic acid sample (Sample 3). Applying this logic filter, the model correctly classifies the sample as containing acetic acid. The acetone, 1,4-dioxane, and vinyl acetate validation samples are not consistent with acetic acid. The results also show good agreement between the predicted and actual acetic acid concentration. Applying the other analyte-specific algorithms for acetone, 1,4-dioxane, and vinyl acetate to the same validation sample set, along with filters equivalent to that used for acetic acid, similar prediction results are observed. Acetone (Figure 11b), 1,4-dioxane (Figure 11c), and vinyl acetate (Figure 11d) samples are correctly identified as containing acetone (Sample 4), 1,4-dioxane (Sample 1), or vinyl acetate (Sample 2), respectively, and there is good agreement between the predicted and actual analyte concentrations.

**Figure 12.**
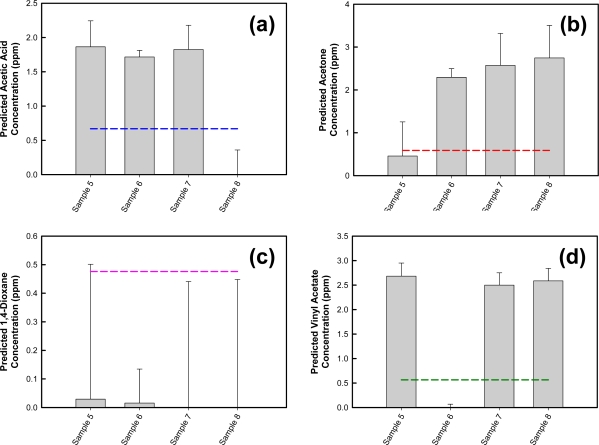
Identification of mixture samples using **(a)** acetic acid-specific algorithm; **(b)** acetone-specific algorithm; **(c)** 1,4-dioxane; and **(d)** vinyl acetate-specific algorithm. Classification for each analyte is based on 4 PCs. The threshold concentration for each analyte is represented by a dotted line.

To further evaluate the performance of the model and the ability of this QCL-based miniaturized photoacoustic sensing platform for the simultaneous detection and discrimination of numerous molecules of interest, mixtures composed of known concentrations of acetic acid, acetone, and vinyl acetate (two or more species in each mixture) were used to challenge the model (Table 4, Samples 5–8). The 1,4-dioxane permeation tube requires a much higher calibration oven temperature compared to the other permeation sources. Therefore, 1,4-dioxane was not included in any of the mixture samples. Prediction results are provided in Figure 12.

As shown in Figure 12a, the acetic acid-specific algorithm applied to this sample set predicts acetic acid concentrations greater than the threshold value for Samples 5, 6 and 7. Applying this logic filter, the model correctly classifies these samples as containing acetic acid. Sample 8 is not consistent with acetic acid. The results also show good agreement between the predicted and actual acetic acid concentrations. Applying the other analyte-specific algorithms for acetone, 1,4-dioxane, and vinyl acetate to the same mixture samples, along with filters equivalent to that used for acetic acid, similar prediction results are observed. Samples containing acetone (Figure 12b) and vinyl acetate (Figure 12d) are correctly identified as containing acetone and/or vinyl acetate, respectively, and there is good agreement between the predicted and actual analyte concentrations. The 1,4-dioxane-specific algorithm predicts concentration values below the model’s threshold concentration with large deviations (Figure 12c), suggesting the absence of 1,4-dioxane in these mixture samples.

Consolidated in Table 4 are results from Figures 11 and 12. The predictions made by the analyte-specific algorithms for acetic acid, acetone, 1,4-dioxane, and vinyl acetate are shown. For each sample, the actual and predicted analyte concentrations are provided. It is important to consider the average error to be expected with these predictions (2 × RMSEP). The predicted concentrations for each analyte fall within the precision of the model (excluding vinyl acetate in Samples 7 and 8). To some extent, deviations of the predicted concentration values from the actual concentration values may be a result of the precision of the permeation tube and/or the calibration generator ovens. Given the current amount of training data and the variance suggested by the model, this level of prediction was expected. An increase in the number of training samples and the range of concentration values within this sample set may result in an improved model. Nonetheless, the results presented in Table 4 demonstrate the ability of this model to accurately discriminate between species having similar molecular components. The analyte-specific algorithms can be used to identify samples that contain acetic acid, acetone, 1,4-dioxane, and/or vinyl acetate, along with samples which are lacking a particular analyte of interest. Furthermore, when samples containing unfamiliar analytes (*i.e.*, analytes not included in the model development) are used to challenge the model, the analyte-specific algorithms predict concentration values with very large uncertainty limits (software-derived deviations). This is a result of the sample data being very different from the training data, suggesting the absence of the analytes of interest (results not shown).

## 4. Conclusions

We have successfully demonstrated a QCL-based MEMS-scale photoacoustic sensing platform combined with a PLS2 chemometrics identification model for trace vapor detection and discrimination. Our results illustrate that this method can be used to distinguish among acetic acid, acetone, 1,4-dioxane, and vinyl acetate based on the laser photoacoustic spectra, which exhibit known absorbance features in the wavelength tuning range of the QCL. We have validated the developed identification algorithm using unknown samples. These validations suggest that this approach can be made to have specificity for selected analytes. To our knowledge, this is the first reported study detailing the use of photoacoustic spectroscopy, employing a single, continuously tunable QCL and a MEMS-scale photoacoustic cell, for the simultaneous detection and molecular discrimination of numerous molecules of interest. The availability of continuously tunable QCLs having a broad wavelength tuning range makes this an attractive approach for a variety of applications, including industrial and military operations.

## Figures and Tables

**Table 1.** Summary of relevant airborne exposure limits. The threshold limit value (TLV) for chemical substances is defined as a concentration in air, typically for inhalation or skin exposure.

**Analyte**	**NIOSH Threshold Limit Value**
Acetic Acid	10 ppm (TWA)^1^
Acetone	250 ppm^2^
1,4-Dioxane	1 ppm (C)^3^
Vinyl Acetate	4 ppm (CL)^4^

1 TWA - Time Weighted Average, 8 h exposure

2 Value averaged over 10 h

3 C - Ceiling, 20 min exposure

4 C - Ceiling, 15 min exposure

**Table 2.** Percent variance for the PLS2 model.

**Factor #**	**Percent Concentration Variance**		**Percent Spectral Variance**	
	Each Factor	Cumulative	Each Factor	Cumulative
1	35.20	35.20	91.14	91.14
2	26.76	61.96	7.43	98.57
3	12.60	74.56	1.28	99.85
4	24.58	99.14	0.13	99.98
5	0.25	99.39	0.01	99.99
6	0.23	99.62	0.01	100.00
7	0.15	99.77	0.00	100.00
8	0.08	99.85	0.00	100.00
9	0.06	99.91	0.00	100.00
10	0.02	99.93	0.00	100.00

**Table 3.** Regression diagnostics for the PLS2 model based on four PCs.

**Analyte**	**Slope**		**Offset**		**R^2^**		**RMSEC**	**RMSEP**
	Calibration	Prediction	Calibration	Prediction	Calibration	Prediction		
Acetic Acid	0.9911	0.9872	0.0036	0.0051	0.9911	0.9899	0.1216	0.1286
Acetone	0.9798	0.9864	0.0127	0.0077	0.9798	0.9773	0.2568	0.2711
1,4-Dioxane	0.9933	0.9877	0.0045	0.0066	0.9933	0.9922	0.1494	0.1615
Vinyl Acetate	0.9984	1.0024	0.0012	−0.0012	0.9984	0.9982	0.0834	0.0905

**Table 4.** PLS2 model predictions for acetic acid, acetone, 1,4-dioxane, and vinyl acetate validation and mixture samples based on 4 PCs. Positive analyte identifications are highlighted.

**Sample #**	**Component Actual Concentration (ppm)**				**Acetic Acid Algorithm Prediction**	**Acetone Algorithm Prediction**	**1,4-Dioxane Algorithm Prediction**	**Vinyl Acetate Algorithm Prediction**
	Acetic Acid	Acetone	1,4-Dioxane	Vinyl Acetate	Concentration (ppm)	Concentration (ppm)	Concentration (ppm)	Concentration (ppm)
1	0.000	0.000	1.590	0.000	0.069	0.166	1.746	0.030
2	0.000	0.000	0.000	1.131	0.000	0.000	0.000	1.060
3	1.114	0.000	0.000	0.000	1.046	0.215	0.050	0.016
4	0.000	1.566	0.000	0.000	0.055	1.146	0.114	0.061
5	1.671	0.000	0.000	2.827	1.864	0.457	0.029	2.683
6	1.671	2.350	0.000	0.000	1.716	2.294	0.016	0.000
7	1.671	2.350	0.000	2.827	1.824	2.573	0.000	2.500
8	0.000	2.350	0.000	2.827	0.000	2.747	0.000	2.586
